# The price of progress: Assessing the financial costs of HIV/AIDS management in East Africa

**DOI:** 10.1097/MD.0000000000042300

**Published:** 2025-05-02

**Authors:** Chioma Vivian Madu, Esther Ugo Alum, Henry Egi Aloh, Okechukwu P. C. Ugwu, Emmanuel Ifeanyi Obeagu, Daniel Ejim Uti, Simeon Ikechukwu Egba, Chris U. A. Ukaidi, Nnachi Benedict Alum

**Affiliations:** a Department Educational Foundations, University of Nigeria, Nsukka, Nigeria; b Department of Research and Publications, Kampala International University, Kampala, Uganda; c Health Economics and Policy Research Unit, Directorate of Health Services, Alex Ekwueme Federal University Ndufu-Alike, Abakaliki, Ebonyi State, Nigeria; d Department of Medical Laboratory Science, Kampala International University, Kampala, Uganda; e Department of Biochemistry, Faculty of Basic Medical Sciences, Federal University of Health Sciences, Otukpo, Benue State, Nigeria; f College of Economics and Management, Kampala International University, Kampala, Uganda.

**Keywords:** antiretroviral therapy, comorbidity, financial burden, healthcare infrastructure, HIV/AIDS, public health

## Abstract

The epidemic of HIV/AIDS, also known as human immunodeficiency virus/acquired immunodeficiency syndrome (HIV/AIDS), continues to be a major global public health concern with significant economic ramifications. East Africa is one of the severely hit regions. The East African economy suffers hugely due to the cost emanating from management of HIV infection. This review offers a thorough analysis of the financial burden of HIV/AIDS treatment, including direct medical costs, infrastructure costs for healthcare, social and indirect costs, and long-term sustainability difficulties. By examining epidemiological statistics, trends in healthcare spending, and socioeconomic factors, we clarify the complex financial environment surrounding HIV/AIDS. The paper also looks at ways to deal with these issues, highlighting the value of cooperation between governments, medical professionals, civil society organizations, and the global community. A thorough literature search that involved a wide range of credible sources, including academic databases (like PubMed, Google scholar, Science Direct, JSTOR), research repositories, government reports, and publications from nongovernmental organizations. It is possible to lessen the economic cost of HIV/AIDS and enhance results for impacted people and communities by making investments in prevention, treatment, and support services while addressing underlying structural concerns.

## 1. Introduction

One of the biggest public health issues of the modern age is the human immunodeficiency virus/acquired immunodeficiency syndrome (HIV/AIDS) epidemic. HIV is a virus that primarily spreads through sexual intercourse, sharing sharp objects like razor blades and needles, or from an infected mother to her fetus.^[1,2]^ HIV infection weakens the body’s immune system, increasing susceptibility to certain infections and the development of particular malignancies, such as cervical cancer.^[3,4]^ Since its inception in the early 1980s, millions of people worldwide have been impacted by HIV infection. HIV impairs immune function, leaving a person more susceptible to infections and diseases. The advanced stage of HIV infection is known as AIDS.^[5–8]^ In 2022, there were about 39 million HIV-positive individuals in the world. Of them, 1.5 million were children under the age of 15, and 37.5 million were adults. Furthermore, 53% of the population was female.^[9]^ Devastating social, economic, and health effects have been caused by the pandemic, especially in sub-Saharan Africa, where HIV/AIDS prevalence is highest.^[8,10]^ But it has also had an impact on communities all around the world, affecting them regardless of their location, age, gender, or social standing. Key populations mostly affected by the virus are men having sex with men, transgender women, sex workers, women and girls trafficked, and injectable drug users. Migrant laborers, those with disabilities, occupants of enclosed places, and female partners of critical populations are also among the vulnerable groups.^[11]^ Significant advancements in HIV/AIDS prevention, treatment, and care have resulted from these efforts. With the advent of antiretroviral medication (ART), HIV was previously considered fatal but is now a chronic illness that many people can manage.^[12]^

Transmission rates have been lowered with the use of preventive measures including education, condom use, and harm reduction initiatives. Historically, attempts to prevent HIV have concentrated on the ABCs: condom usage, faithfulness, and abstinence.^[13]^ ART has reduced the rate of infection and deaths linked to HIV/AIDS, converting the virus from an acute infection with high mortality to a chronic, treatable illness. Individuals living with HIV (PLWH) are experiencing longer life expectancies,^[14]^ and as they get older, they are frequently more vulnerable to negative health outcomes than the general population because they are developing more chronic comorbidities such depression, type 2 diabetes, and hypertension.^[15,16]^ According to a study, comorbidities and concurrent medication use are more common in Medicaid-insured individuals under 65, and these trends increase with age.^[17]^ According to a study, compared to non-HIV controls, people with HIV and comorbidities have greater overall healthcare expenses as well as higher costs for pharmacy, outpatient care, and healthcare utilization.^[18]^

Research in East Africa has highlighted the potential for integrating hypertension care into HIV care, with minimal additional costs.^[19]^ This approach has been found to be financially feasible and associated with positive clinical outcomes, including improved retention rates and viral load suppression.^[20,21]^ However, the cost-effectiveness of specific interventions varies, with preventive measures and tuberculosis treatment being particularly cost-effective.^[22]^ These findings underscore the importance of considering the economic implications of different strategies for managing comorbidities in HIV patients in East Africa.

The enormous expense of HIV/AIDS care is a burden on East Africa’s economy. In 2019, HIV management costs in Kenya and Malawi amounted to US$6175,960 and US$4261,207 respectively, with clinical services, management, peer outreach, and monitoring being the most expensive areas.^[23,24]^

Significant progress was made in HIV care in East Africa between 2015 and 2023, with the enrollment of 2937 PLWH, 95.6% of whom initiated ART.^[25–27]^ This progress is in line with the global trend, where ART coverage has increased in the 10 highest burden HIV countries in Africa.^[18,28–30]^ However, challenges persist, particularly concerning missed clinic visits.^[25]^ These challenges are consistent with the global context, where late diagnosis, poor linkage to care, and ART interruptions contribute to “excess” deaths among PLWH.^[25]^ To address these challenges, community-based models and mobile health technology have been implemented to increase ART coverage and retention.^[28,31]^ Despite these efforts, the need for further improvement in HIV care is evident, as highlighted by the suboptimal treatment of HIV-2 in West Africa.^[32,33]^

Ward et al^[34]^ reported that other management expenses declined but HIV treatment costs grew in line with expected survival rates in their study. This finding emphasizes the need for better disease management and prevention measures in order to improve outcomes and save costs. The available data indicate a trend towards increases in comorbidities, healthcare resource utilization, and related expenditures among PLWH when taking into account the possible lifetime impact of HIV in individuals who are at risk or infected.^[34]^ Given the advancements in ART, this is probably going to rise as patients survive longer. Pre-exposure prophylaxis, which lowers the chance of HIV infection in high-risk persons, is still underutilized, which adds to the financial burden on HIV patients. It is undervalued how much money HIV management costs and the financial benefits of adequate HIV prevention measures. Thus, this paper highlighted the enormous financial cost of managing HIV, particularly in light of the co-occurring conditions related to HIV. In summary, this paper reiterated the importance of improving preventative tactics and the cost-effectiveness of preventive interventions (Table 1). A comprehensive review of the literature was done using a variety of reliable sources, such as government papers, academic databases (such as PubMed, Google Scholar, Science Direct, and JSTOR), research archives, and publications by non-governmental organizations (NGOs).

**Table 1:** An overview of HIV/AIDS: transmission, impact, treatment advancements, and economic burden.

Public health issue	HIV/AIDS transmission	Impact of HIV/AIDS	Key populations & challenges	Advancements in treatment & care	Economic & healthcare burden	Countries	References
HIV/AIDS remains a major global public health issue	Primarily spreads through sexual contact, sharing of sharp objects, and mother-to-child transmission	Weakened immune system increases susceptibility to infections and cancers like cervical cancer	Key affected populations include MSM, transgender women, sex workers, trafficked women and girls, and injectable drug users	ART has transformed HIV from a fatal disease to a chronic manageable condition	The financial burden of HIV care is substantial, especially in East Africa	Global (with a focus on sub-Saharan Africa)	^[1–5]^
Since the 1980s, millions have been affected globally	Advanced HIV stage is AIDS, leading to increased vulnerability	2022 statistics: 39 million people living with HIV, including 1.5 million children and 37.5 million adults (53% female)	Vulnerable groups also include migrant laborers, people with disabilities, and partners of high-risk individuals	ART, along with condom use, education, and harm reduction, has reduced transmission	HIV care costs in Kenya and Malawi in 2019 were US$6175,960 and US$4261,207, respectively	Kenya, Malawi, Global	^[3,6–8]^
Sub-Saharan Africa has the highest HIV prevalence	Devastating social, economic, and health consequences	Communities worldwide are affected regardless of age, gender, or status	Barriers to care include stigma, late diagnosis, and ART interruptions	PLWH now have longer life expectancies, but with increased chronic comorbidities	Challenges include late diagnoses, poor linkage to care, and ART adherence issues	Sub-Saharan Africa	^[9–11]^
HIV prevention includes ABCs: Abstinence, Being faithful, and Condom use	ART has lowered transmission rates and improved survival	Higher comorbidities in PLWH, including depression, diabetes, and hypertension	Medicaid-insured individuals under 65 show higher healthcare costs	Integrating hypertension care into HIV services is cost-effective in East Africa	ART expansion between 2015 and 2023: 95.6% of 2937 PLWH initiated treatment	East Africa	^[13–16]^
HIV patients have higher outpatient, pharmacy, and healthcare utilization costs	Financial burden on healthcare systems is increasing as PLWH live longer	Pre-exposure prophylaxis (PrEP) is underutilized, adding to the burden	Cost-effectiveness of tuberculosis treatment and prevention strategies varies	Mobile health technology and community-based models improve ART retention	ART coverage has improved in Africa’s top 10 high-burden countries	Sub-Saharan Africa	^[19–21]^
Management expenses for HIV increased in parallel with higher survival rates	HIV/AIDS economic burden includes treatment costs and comorbidity management	Studies indicate increasing costs for PLWH due to long-term care needs	Prevention measures remain undervalued despite potential financial benefits	Treatment and care costs continue to rise, emphasizing the need for better prevention	HIV-2 treatment in West Africa remains suboptimal despite global ART progress	West Africa	^[26–28]^

ART = antiretroviral therapy, MSM = men having sex with men, PLWH = people living with HIV.

## 2. Methodology/search strategy

*Defined search terms*: Identified keywords related to the topic such as HIV/AIDS; Financial burden; Public health; antiretroviral therapy; Comorbidity; Healthcare infrastructure were used.

Select *databases*: Appropriate academic databases like PubMed, Google Scholar, Web of Science, Science Direct, JSTOR, research repositories, government reports, and publications from NGOs were searched.

### 2.1. Inclusion criteria

*Geographic location*: Only studies conducted in East African countries such as Kenya, Uganda, Tanzania, Rwanda, Burundi, and South Sudan were used.

*Time frame*: Studies conducted within a certain time period relevant to the current situation of HIV/AIDS management in East Africa, basically, between 2015 to 2024.

*Study design:* Research studies that provide relevant data on the financial costs of HIV/AIDS management, including quantitative studies, qualitative studies, and mixed-methods studies.

*Language*: Studies published in English or translated into English if relevant data are available.

*Outcome measures*: Studies that report on the financial burden of HIV/AIDS management, including direct medical costs, indirect costs (such as lost productivity), and out-of-pocket expenses for patients.

### 2.2. Exclusion criteria

This study excluded studies involving participants outside the specified region or not related to HIV/AIDS, lacks original data on financial costs, focuses solely on treatment efficacy, lacks transparency, lacks adequate methodology, is non-English, and conducted in settings different from East Africa. The review process involved searching, data extraction, synthesis, critical appraisal, writing, peer review, and finalizing the manuscript based on feedback.

### 2.3. The financial cost of HIV/AIDS management in East Africa

The cost of treating HIV/AIDS is a complex problem with broad ramifications for people, communities, healthcare systems, and economies all around the world. Although medical research has made HIV/AIDS from a potentially fatal illness to a chronic illness that can be managed, the costs of treatment are still high and provide difficulties on many fronts.

### 2.4. Direct medical expenses for diagnostic, monitoring services, and healthcare infrastructure

The direct expenditures of medical care are the main financial burden of HIV/AIDS therapy. Included in this are costs associated with antiretroviral medication (ART), the mainstay of HIV treatment. With the use of a combination of drugs known as ART, people with HIV can live longer, healthier lives. These drugs can be expensive, though, especially in low- and middle-income nations where access to reasonably priced healthcare is scarce. The type of drug, intricacy of the regimen, and geographic location are some of the variables that affect the cost of ART.^[35]^ In a recent study by McBain et al^[36]^, HIV/AIDS service delivery costs in Uganda varied from US$ 8.18 for HIV testing and counseling to US$ 43.43 for ART (for clients in whom HIV was suppressed). In Tanzania, the prices for elective male medical circumcision ranged from US$ 28.00 to US$ 3.67 per visit for HIV testing and counseling. Consumables accounted for over 60% of expenditures in both countries, making them the primary cause of cost increases. In Kenya and Malawi, the total economic expenses of HIV management in 2019 were US$6175,960 and US$4261,207, respectively. Similarly, Titou et al^[37]^ reported that Morocco had a mean expenditure per HI-positive patient of 24.2 euros. Clinical services, management, peer outreach, and monitoring and data utilization were the most expensive program areas. In Kenya, the average cost per contact was $127, whereas in Malawi, it was $279.^[23]^ In the financial year 2022/2023 in Rwanda, the Global Fund (for AIDS, TB and Malaria) contributed US Dollars (USD) 72,305,520, while the US Government, Rwanda, and UN contributed USD 67,884,250, 24,839,743, and 708,213, respectively. In terms of spending, the Government of Rwanda spent USD 21,479,042, the US government spent USD 67,884,250, and the Global Fund spent USD 51,723,894. Finally, the UN spent USD 708,213 for. This amounts to USD 141,516,577, or 86% of the budget, for the HIV management in Rwanda for the year 2022/2023.^[38]^ In Rwanda, the average cost per client diagnosed with HIV-positive ranged from US$119 to 122 in Zambia and US$1367 to 2093 in Rwanda during the 2011 to 2012 period. The cost per client tested for HIV ranged from US$5 to 7 in Rwanda. The mean expense per patient for PMTCT testing varied between US$18 and US$20 in Rwanda. In Rwanda, the average cost of antiretroviral prophylaxis per client was from US$2314 to 3204, whereas the average cost of nevirapine per baby was between US$2359 to 3257.^[39]^

Apart from the costs of medication, there are other charges related to HIV testing, illness monitoring, and managing related comorbidities. It is crucial to regularly monitor the viral load, CD4 cell count, and other biomarkers in order to evaluate the effectiveness of treatment and the course of the disease. Medical consultations, laboratory services, and diagnostic testing all add to the overall cost of HIV/AIDS care.^[40]^ A robust healthcare infrastructure, comprising hospitals, clinics, laboratories, and skilled healthcare workers, is necessary to provide comprehensive HIV/AIDS care.^[41]^ Such infrastructure is expensive to build and maintain, especially in areas with limited resources where healthcare systems might not have the funding. For people living with HIV/AIDS to have access to high-quality care and treatment services, infrastructure development must be funded.^[42]^

### 2.5. Social and indirect costs

In addition to the direct costs of healthcare, there are a number of indirect expenditures related to HIV/AIDS treatment that can increase the financial burden. These include lost productivity as a result of disability, illness, and early death in HIV/AIDS-affected individuals. When a patient’s sickness advances to a more advanced state or when their drug side effects make it difficult for them to work, the illness and its treatment may result in decreased productivity, unemployment, and absenteeism from work.^[43]^ HIV/AIDS has significant social ramifications in addition to health effects, which add to the disease’s financial impact. Affected people may experience social isolation, mental health problems, and a reduction in their quality of life as a result of the stigma and discrimination surrounding the illness. These social costs can take many different forms, such as lower educational achievement, less work possibilities, and strained social connections, all of which have an impact on society and the economy on an individual basis.^[44]^

### 2.6. Effects on families and support staff

HIV/AIDS has a financial cost that affects not only the infected person but also their family members and carers. The financial burden of providing care for sick family members frequently falls on family members. This includes charges for transportation, assistance with medication adherence, and modifications to the home to meet the needs of the sick person. When the main provider for the family is afflicted with HIV/AIDS, the loss of income can intensify already-existing socioeconomic disparities and drive families into deeper poverty.^[45]^

### 2.7. Challenges with healthcare financing

The financing of HIV/AIDS therapy presents major obstacles for foreign assistance organizations, governments, and healthcare providers. For individuals in need to always have access to care and treatment, long-term financing sources are required. Nevertheless, it might be difficult to raise enough money for HIV/AIDS programs due to conflicting healthcare priorities, financial limitations, and donor reliance, especially in low-resource areas where the disease burden is greatest.^[46]^ Economic difficulties are made worse by disparities in access to HIV/AIDS treatment, especially for underprivileged and marginalized groups. Socioeconomic variables that can make it difficult to obtain healthcare services and stick to treatment plans include poverty, illiteracy, remote locations, and discrimination based on sexual orientation, gender, or race.^[34]^ Targeted interventions that enhance care accessibility, lessen stigma, and address underlying social determinants of health are necessary to overcome these discrepancies.

### 2.8. Long-term sustainability

The long-term viability of healthcare systems becomes crucial as more individuals with HIV/AIDS receive treatment and live longer with the illness. The number of people using ART is increasing, which puts more strain on healthcare resources like drug supply chains, lab spaces, and medical staff. A comprehensive strategy that takes into account not just the short-term funding requirements but also the long-term sustainability and scalability of treatments is needed to address the financial burden of HIV/AIDS treatment. In order to effectively address the complicated and varied issue of the financial burden of HIV/AIDS treatment, governments, healthcare providers, civil society organizations, and the international community must work together. A complete solution to the economic issues faced by HIV/AIDS must include investments in healthcare facilities, attempts to decrease stigma and prejudice, initiatives targeted at addressing underlying social determinants of health, and sustainable finance.^[47]^ It is possible to lessen the financial burden of HIV/AIDS and enhance the lives of the millions of people who are afflicted by this illness by strengthening equity, investing in preventative initiatives, and giving priority to access to reasonably priced treatment and care services.

### 2.9. Strategies carried out to improve late diagnosis and access to antiretroviral treatment

East Africa needs to improve late diagnosis and access to ART through a multimodal approach that includes government policy, community participation, healthcare systems, and international cooperation. The following are some tactics that are frequently used and how they affect the reduction of HIV-related morbidity and mortality^[48–51]^:

*Increased testing and early diagnosis*: Early diagnosis can result from implementing large-scale HIV testing programs, especially those aimed at high-risk populations, and incorporating HIV testing into standard healthcare services. Mobile clinics, home-based testing, and community-based testing have all shown promise in reaching underserved or distant areas.

### 2.10. Strengthening

*Healthcare infrastructure*: In order to guarantee prompt access to HIV testing and treatment, it is imperative to invest in healthcare infrastructure, which includes clinics, laboratories, and supply chains. As part of this, transportation networks should be improved to make it easier to distribute medications to rural areas.

*Task shifting and training*: Educating healthcare professionals who are not doctors to do HIV testing, counseling, and ART delivery eases the strain on overworked healthcare systems and enhances access to care, particularly in underprivileged areas. Examples of these professionals include nurses and community health workers.

*Reducing stigma and discrimination*: By addressing stigma and discrimination related to HIV/AIDS via advocacy, community outreach, and education, more people may get tested and receive treatment without worrying about the consequences to their social standing.

*Integration of HIV services*: This can enhance access to comprehensive care and raise the chance of an early diagnosis and start of treatment. It can also be integrated with other healthcare services, such as TB treatment, maternity and child health, and sexual and reproductive health services.

*Support from policy and regulation*: An atmosphere that is supportive of HIV prevention and treatment programs is created by enacting laws that guarantee confidentiality, safeguard the rights of individuals living with HIV/AIDS, and encourage HIV testing, treatment, and care.

*Peer support and community engagement*: Including communities in HIV prevention, testing, and treatment initiatives increases service uptake, builds trust, and gives those living with HIV/AIDS access to peer support networks. In order to provide information, counseling, and adherence support, community health workers, peer educators, and support groups are essential.

*International coordination and finance*: Effective HIV/AIDS programs must be implemented through cooperation between governments, international agencies, NGOs, and donors in order to mobilize resources and share best practices. International funding sources can fill in funding gaps for HIV prevention, testing, and treatment programs.

### 2.11. Comparative analysis of HIV/AIDS management in resource-limited regions: ART usage, costs, and outcomes

This comparative examination of HIV/AIDS management in resource-constrained areas such as Sub-Saharan Africa, Latin America, and Eastern Europe elucidates the issues encountered by these locations. The use of ART is substantial but constrained, with donor financing being pivotal for accessibility.^[52–54]^ Latin America has extensive availability of ART; nonetheless, discrepancies remain between urban and rural regions.^[55–58]^ Eastern Europe provides access to ART; nonetheless, marginalized populations often encounter obstacles stemming from stigma and prejudice.^[59–61]^ The economic cost of HIV/AIDS management differs across various locations. In Sub-Saharan Africa, antiretroviral therapy programs are mostly financed by donors, which guarantees accessibility but presents sustainability issues.^[62]^ Latin American nations depend on public financing; yet, economic crises affect both affordability and distribution. Eastern Europe encounters elevated ART expenses owing to restricted availability of generic medications, rendering therapy less accessible for at-risk individuals.^[63]^

Compliance rates differ by geography, with modest levels in Sub-Saharan Africa attributable to socioeconomic obstacles and healthcare accessibility challenges. Latin America typically exhibits moderate to high compliance rates; nonetheless, rural regions have challenges. Eastern Europe exhibits low to moderate compliance, profoundly influenced by stigma and prejudice against those living with HIV/AIDS. Mortality rates attributable to HIV/AIDS continue to be a problem, particularly in Sub-Saharan Africa, but they are declining owing to the expansion of antiretroviral therapy programs. Latin America has a modest mortality rate, with substantial declines credited to robust public health programs. Eastern Europe has elevated mortality rates, especially among high-risk demographics, intensified by delayed diagnosis and the commencement of treatment. To improve HIV/AIDS management, each area must confront current obstacles. Sub-Saharan Africa need to prioritize the enhancement of domestic pharmaceutical manufacturing and the augmentation of healthcare accessibility. Latin America needs to enhance rural healthcare infrastructures and provide continuous financial support. Eastern Europe must address stigma, enhance accessibility, and augment the supply of inexpensive generic antiretroviral therapy drugs. Table 2 summarizes these comparisons.

**Table 2:** Comparative analysis of HIV/AIDS management in resource-limited regions: ART usage, costs, and outcomes.

S/N	Region	ART usage	Financial cost	Compliance rate	Success rate	Mortality rate	Major success achieved	Future Direction	References
1	Sub-Saharan Africa	High but limited by availability	Mostly donor-funded, variable affordability	Moderate (due to adherence challenges)	Improving with better regimens	High but decreasing	Scale-up of ART programs, reduction in mother-to-child transmission	Expansion of local drug production, better healthcare access	^[52–54,64]^
2	Latin America	Widespread but disparities exist	Public funding available, but economic crises impact affordability	Moderate to high, but disparities exist	High in urban areas, lower in rural areas	Moderate	Strong public health programs, good urban ART success	Strengthening rural healthcare access, increasing funding	^[55–58]^
3	Eastern Europe	Available but limited in marginalized groups	High cost due to limited generic drugs	Low to moderate, affected by stigma and discrimination	Variable, better in wealthier regions	High in high-risk populations	Improved diagnostics and treatment programs	Reducing stigma, improving accessibility, increasing generic drug production	^[59–61]^

ART = antiretroviral therapy.

## 3. Conclusion

The substantial financial cost related to HIV/AIDS treatment and management in East Africa has been made clear by this manuscript. By investigating direct medical expenditures, the costs of the healthcare infrastructure, the social ramifications, and the funding constraints, we have shown the convoluted and diverse nature of the financial difficulties this pandemic presents. The financial burden on people, families, healthcare systems, and economies around the world continues to increase despite major improvements in HIV/AIDS treatment, such as the introduction of ART. These advancements are exacerbated by discrepancies in access to care and continuing social stigmas. Governments, healthcare providers, civil society organizations, and the global community must work together to address these issues. A comprehensive response must include targeted activities to decrease stigma and discrimination, sustainable financing methods, and investments in healthcare infrastructure. Millions of people in East Africa infected by HIV/AIDS can have their quality of life improved and the financial burden of the disease can be lessened by placing a high priority on equity, access to care, and prevention measures.

## Author contributions

**Conceptualization:** Chioma Vivian Madu, Esther Ugo Alum, Henry Egi Aloh, Daniel Ejim Uti.

**Data curation:** Chioma Vivian Madu, Henry Egi Aloh.

**Methodology:** Emmanuel Ifeanyi Obeagu, Simeon Ikechukwu Egba.

**Resources:** Okechukwu P. C. Ugwu.

**Supervision:** Daniel Ejim Uti.

**Validation:** Esther Ugo Alum, Henry Egi Aloh, Chris U. A. Ukaidi, Nnachi Benedict Alum.

**Writing – original draft:** Chioma Vivian Madu, Daniel Ejim Uti.

**Writing – review & editing:** Chioma Vivian Madu, Esther Ugo Alum, Henry Egi Aloh, Okechukwu P. C. Ugwu, Emmanuel Ifeanyi Obeagu, Daniel Ejim Uti, Simeon Ikechukwu Egba, Chris U. A. Ukaidi, Nnachi Benedict Alum.
